# The Pentraxins 1975–2018: Serendipity, Diagnostics and Drugs

**DOI:** 10.3389/fimmu.2018.02382

**Published:** 2018-10-16

**Authors:** Mark. B. Pepys

**Affiliations:** ^1^Wolfson Drug Discovery Unit, Centre for Amyloidosis and Acute Phase Proteins, University College London, London, United Kingdom; ^2^National Institute for Health Research University College London Hospitals Biomedical Research Centre, London, United Kingdom

**Keywords:** pentraxin, C-reactive protein, serum amyloid P component, amyloidosis, drugs, miridesap, dezamizumab, complement

## Abstract

The phylogenetically ancient, pentraxin family of plasma proteins, comprises C-reactive protein (CRP) and serum amyloid P component (SAP) in humans and the homologous proteins in other species. They are composed of five, identical, non-covalently associated protomers arranged with cyclic pentameric symmetry in a disc-like configuration. Each protomer has a calcium dependent site that mediates the particular specific ligand binding responsible for all the rigorously established functional properties of these proteins. No genetic deficiency of either human CRP or SAP has been reported, nor even any sequence polymorphism in the proteins themselves. Although their actual functions in humans are therefore unknown, gene deletion studies in mice demonstrate that both proteins can contribute to innate immunity. CRP is the classical human acute phase protein, routinely measured in clinical practice worldwide to monitor disease activity. Human SAP, which is not an acute phase protein, is a universal constituent of all human amyloid deposits as a result of its avid specific binding to amyloid fibrils of all types. SAP thereby contributes to amyloid formation and persistence *in vivo*. Whole body radiolabelled SAP scintigraphy safely and non-invasively localizes and quantifies systemic amyloid deposits, and has transformed understanding of the natural history of amyloidosis and its response to treatment. Human SAP is also a therapeutic target, both in amyloidosis and Alzheimer's disease. Our drug, miridesap, depletes SAP from the blood and the brain and is currently being tested in the DESPIAD clinical trial in Alzheimer's disease. Meanwhile, the obligate therapeutic partnership of miridesap, to deplete circulating SAP, and dezamizumab, a humanized monoclonal anti-SAP antibody that targets residual SAP in amyloid deposits, produces unprecedented removal of amyloid from the tissues and improves organ function. Human CRP binds to dead and damaged cells *in vivo* and activates complement and this can exacerbate pre-existing tissue damage. The adverse effects of CRP are completely abrogated by compounds that block its binding to autologous ligands and we are developing CRP inhibitor drugs. The present personal and critical perspective on the pentraxins reports, for the first time, the key role of serendipity in our work since 1975. (345 words)

## Discovery of the pentraxins

When I returned to clinical training at the Royal Postgraduate Medical School in London in 1973, after my PhD discovery of the role of complement in induction of antibody formation (1–5), the Head of Medicine, Professor (later Sir) Christopher Booth, advised to me to start a more clinical research project. He suggested that I should “crack Crohn's disease.” This led me serendipitously^1^ to the pentraxins.

In the early 1970s, reduced numbers of circulating T cells had been reported in many chronic inflammatory diseases of unknown etiology, including Crohn's disease. Although this was actually an artifact caused by differential loss of T cells during isolation of peripheral blood lymphocytes (6), T cell function in Crohn's disease was still of interest in 1975 when Henry Gewurz reported that C-reactive protein (CRP) bound to antigen activated T cells and suppressed their functions (7). I assumed that CRP production would be increased in active Crohn's disease and speculated that it could be responsible for suppression of T cell function. However, to my surprise, in 1975, CRP measurements had not been reported in either Crohn's disease or ulcerative colitis and I set out to do this for the first time.

There were no commercial quantitative immunoassays for CRP at that time. I therefore isolated some human CRP and immunized a rabbit to raise my own anti-CRP antiserum. This “famous” rabbit, known only as R1032, produced strong precipitating antibodies to CRP, which were excellent for electroimmunoassay. But it also produced precipitating antibodies against another, immunochemically distinct, normal trace plasma protein with fast α-mobility, which was not an acute phase reactant. None of the available antisera to known human plasma proteins reacted with this unknown protein; which I designated “protein X.” I had neither the resources nor the motivation to attempt amino acid sequencing and, since many plasma proteins had not been sequenced, it might not have helped. Indeed, as it transpired, if we had sequenced it then it would have been the first time for that protein! Meanwhile I used the antiserum to assay CRP concentrations in clinical samples using electroimmunoassay (8), and made important new observations (see section Routine clinical measurement of CRP), whilst ignoring the immunoprecipitates produced by the contaminating antibodies to protein X.

**Figure 1:**
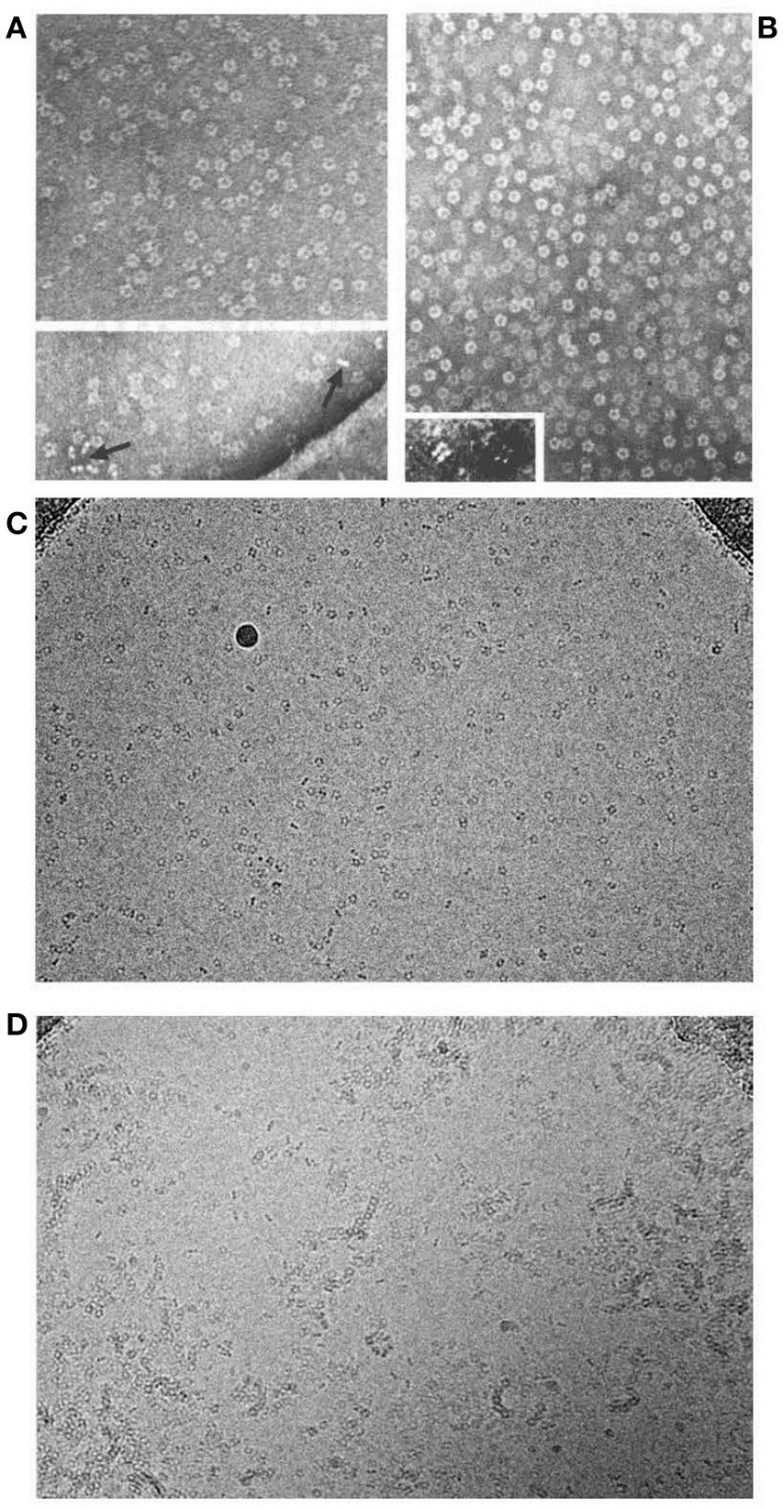
Molecular appearance of the pentraxins. **(A)** Negatively stained electron microscopic image of human CRP with the characteristic symmetrical pentameric ring viewed face on. Inset shows disc-like appearance of single molecules side on. **(B)** Negatively stained electron microscopic image of human SAP with the characteristic symmetrical pentameric ring viewed face on. Inset shows typical face to face double pentamer forming the decameric assembly present in calcium free conditions. This was thought to be the normal assembly of human SAP until the actual physiological native pentameric structure was demonstrated (15). **(C)** Cryoelectron microscope image of our preparations of human CRP (mass 115,135) and of **(D)** human SAP (mass 127,310) showing the actual native pentraxin structure with no staining or artefactual enhancement (courtesy of Dr Richard Henderson).

My CRP antigen preparation had obviously been contaminated with protein X and I therefore sought to improve the isolation procedure. CRP was named for its calcium dependent binding to pneumococcal C-polysaccharide so calcium dependent affinity chromatography was an obvious and attractive possibility (9). CRP from whole serum bound efficiently, in the presence of calcium, to suitable ligands that had been covalently immobilized on Sepharose, commercial beaded agarose, and other serum proteins were then washed away. The CRP could then be eluted by calcium chelation but, regardless of the immobilized ligand, protein X was still present. The obvious control experiment showed that, unlike CRP, protein X underwent avid calcium dependent binding to plain unsubstituted Sepharose, and was eluted by calcium chelation. This simple one step isolation in pure form of a trace plasma protein was unique and demanded identification of protein X. In collaboration with Arnold Feinstein and Ed Munn, who had first reported negative staining electron microscopy (EM) of IgM, EM of isolated protein X instantly identified it as amyloid P component (AP) (10–14) (Figure 1). Unexpectedly, isolated CRP had a remarkably similar appearance (Figure 1). Both these homopentameric, calcium dependent, ligand binding, plasma proteins were composed of globular subunits arranged with cyclic symmetry in a disc like configuration.

At the same time, two other groups were working on these two proteins. Robert Painter isolated the C1 component of complement from whole serum by calcium dependent affinity chromatography on IgG covalently immobilized on Sepharose (16). In addition to the known subcomponents, C1q, C1r, and C1s, he always found a fourth protein that he designated C1t (17) and which he soon found to resemble AP (18) in the EM. Meanwhile Alex Osmand and Henry Gewurz observed marked *N*-terminal sequence homology between CRP and C1t (AP) and, together with Painter noted their highly characteristic, similar EM appearances (19). Osmand coined the name “pentraxin” for this newly recognized protein family, derived from the Greek words “penta” (five) and “ragos” (berries), representing the EM appearance of the molecules^2^. We confirmed immunochemically that our protein X was serum amyloid P component (SAP) (21), and my discovery of its calcium dependent binding to unsubstituted Sepharose explained its presence in Painter's C1 preparations, showing that it had nothing to do with C1.

Work on the pentraxins, CRP and SAP, then proceeded energetically in various directions, albeit with some false starts. The claims for binding and effects of CRP on lymphocytes, that had serendipitously introduced me to the field, proved not to be reproducible. Indeed there have been, and still are, a number of highly controversial claims about properties, functions and effects of CRP and SAP. However, the early discovery of classical pathway complement activation by CRP following its binding to macromolecular ligands (22, 23) withstood the test of time and it is unequivocally crucial for the role of CRP in exacerbation of tissue damage (24).

## What are pentraxins?

“What's in a name? That which we call a rose

By any other name would smell as sweet.”

William Shakespeare. Romeo and Juliet (II, ii, 1-2)

The question is both scientific and semantic. The neologism, pentraxin, was invented by Alex Osmand (19) from the Greek words meaning five berries, to describe the unique cyclic pentameric symmetrical appearance of the molecules of human CRP and SAP. The appearance is shared by the pentraxins from all the different species that have been visualized, apart from the hexameric CRP homolog of the horseshoe crab, *Limulus polyphemus* (25) and other multimeric invertebrate homologs. In addition to the numbers of subunits there are other differences between species. For example, rat CRP differs from the human CRP in being glycosylated and in having a covalent disulphide bond between one pair of protomers in each pentameric molecule (26). Nevertheless the very high degree of sequence homology, together with the instantly recognizable pentraxin molecular appearance, demonstrate that all the different plasma proteins characterized by calcium dependent binding to the classical pentraxin ligands are unequivocally members of the same family. The “long pentraxins” (27) do not have the pentraxin appearance although they contain a domain with modest sequence homology to pentraxins. Also calcium binding, which is required for stability of the secondary, tertiary and quaternary structures of most actual pentraxins, and is essential for the specific ligand binding that underlies all robustly reproducible pentraxin functions, is not a feature of the “long pentraxins.” An analogous situation exists in relation to the many diverse non-immunoglobulin proteins which contain immunoglobulin sequence homology domains but do not share antibody-like specific epitope binding. They are, accordingly, not called antibodies but the well-established “long pentraxin” names are evidently not going to change.

## Pentraxin structure

**Figure 2:**
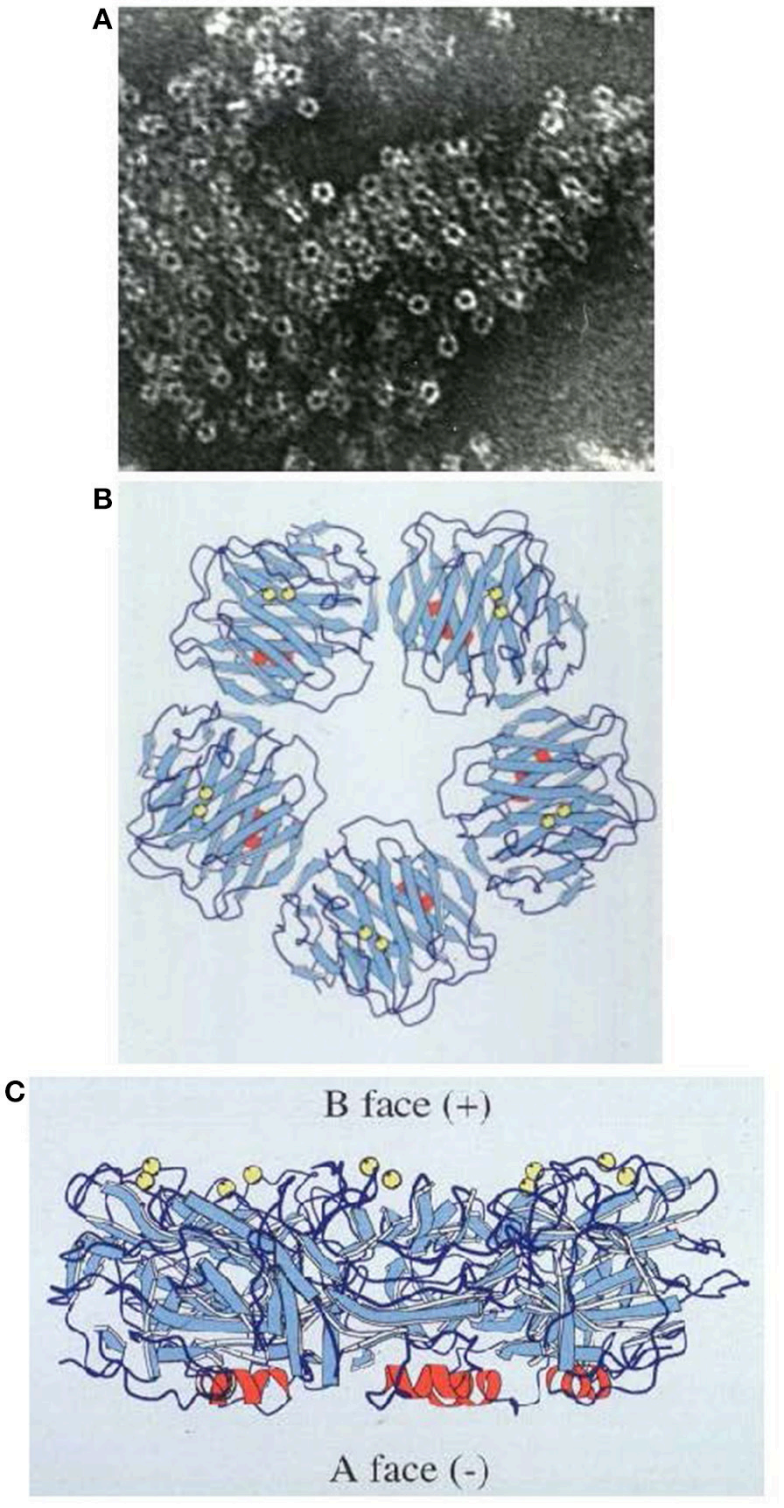
Structure of human SAP. **(A)** Electron micrograph of negatively stained human SAP molecules. **(B)** Ribbon diagram of the 3D X-ray crystal structure of human SAP face on (“B” face uppermost). **(C)** Ribbon diagram of the 3D X-ray crystal structure of human SAP side on. Calcium atoms are represented as yellow spheres located on the binding, “B” face; the single small α-helix of each protomer is shown in red, located on the “A” face (29); β-sheets are in pale blue and loops in dark blue.

**Figure 3:**
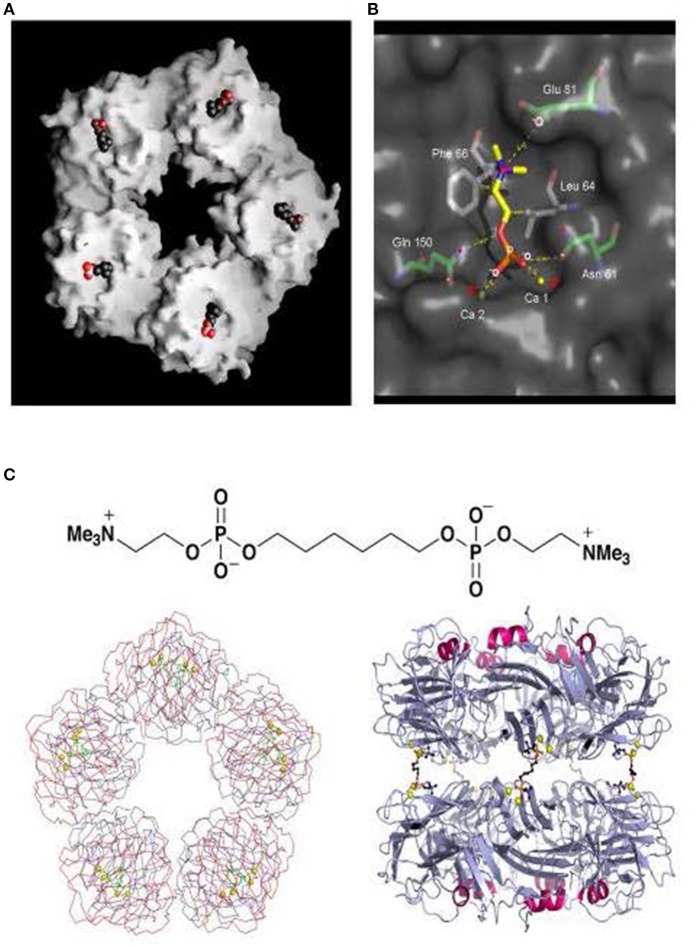
Structure of human CRP with bound phosphocholine and bis(phosphocholine)-hexane. **(A)** Space filling model of “B” face of human CRP with phosphocholine bound in each of the protomer binding sites. **(B)** 3D X-ray crystal structure of phosphocholine in the binding pocket of a single CRP protomer within the native molecule, showing the ligand interactions with calcium and the CRP residues responsible for binding. **(C)** The structure of bis(phosphocholine)-hexane (above) and the structure of the complex formed by two CRP molecules cross linked by five bis(phosphocholine)-hexane molecules; face on (left) and side on (right) [From reference (32) with permission of Macmillan Publishers Ltd].

In 1994, we reported the first pentraxin structure: the 3D X-ray crystal structure of human SAP alone and of its calcium dependent complex with the cyclic pyruvate acetal of galactose (28) (Figure 2). SAP crystallized easily but it followed nearly 17 years of failure to grow reproducible crystals of human CRP suitable for X-ray crystallography. Eventually I thought of lowering the calcium concentration to reduce the solubility of human CRP as it starts to denature. This yielded a batch of poor and fragile crystals that nonetheless provided a low resolution structure of partly calcified CRP (30). Then my serendipitous, inadvertent, “overconcentration” of a batch of isolated human CRP to more than 20 mg/ml in the presence of physiological calcium, caused sudden, concentration dependent, reversible precipitation of the protein that pointed the way to effective crystallization conditions. Finally, the full physiological structure of human CRP alone and with bound phosphocholine was solved (31) (Figure 3).

The tertiary fold of the two human pentraxins is closely similar, with the main chain forming a flattened β-jellyroll with closely tethered loops between the antiparallel strands. There is a short α-helix on one face, the “A” face (29), of each protomer and calcium tethered loops on the opposite, binding, “B,” face, forming the shallow ligand binding pocket. Although there is only about 11% amino acid sequence homology with the human pentraxins, the proteins with the most similar β-jellyroll tertiary fold are the legume lectins, pea lectin and concanavalin A (28). This architecture is apparently an effective support for proteins that provide calcium dependent binding of carbohydrate and other non-protein ligands.

The extensively hydrogen bonded antiparallel β-strands and tightly bound loops make the pentraxins rather resistant to proteolysis but, in the absence of calcium, the calcium coordinating loops are disorganized and readily cleaved (33, 34). Calcium is obviously always present at ~2 mM in the extracellular environment *in vivo* but the normally very stable, albeit non-covalent, native pentameric assembly of human CRP is notably destabilized in the absence of calcium. Free protomers are released and the protein readily aggregates. Use of non-physiological experimental conditions, leading to artefactual properties of human CRP, has produced misleading conclusions about properties and effects of the protein. Even worse, effects caused by sodium azide preservative in CRP preparations (35) and contamination of recombinant CRP by bacterial products (36) have been misleadingly attributed to CRP itself.

Human SAP is also more susceptible to proteolytic cleavage in the calcium coordinating loops when calcium is absent (34) but, unlike human CRP, under these non-physiological conditions human SAP forms stable decameric assemblies of pairs of pentameric SAP molecules interacting “B” face to “B” face (37). The interaction is mediated by displacement of the loop comprising residues 134–151 in each protomer and then binding of the loop in the inter-subunit groove in the “B” face of the apposed pentameric ring (37). For a number of years we believed that this double pentamer was the native state of the SAP molecule [see for example (28)], in contrast to the single pentamer of human CRP. However, careful characterization of the molecular form of native SAP within the milieu of whole serum showed that human SAP is actually a single pentamer that is not complexed with any other plasma constituent (15). These studies are challenging because, as we had discovered very early on, exposure of isolated pure human SAP to calcium leads to rapid autoaggregation (38). Aggregated human SAP acquires novel ligand binding and other properties (39), unfamiliarity with which produced a number of misleading reports on possible functions of SAP. We eventually showed that human SAP autoaggregation is mediated by binding of the exposed γ-carboxylate of residue Glu167 on one SAP molecule in the calcium dependent ligand pocket on another (40). This is prevented by the presence of physiological concentrations of serum albumin (15), probably, at least in part, by virtue of calcium binding by the albumin, critically lowering the free ionisable calcium concentration. In any case, in the presence of the calcium, that is required for its ligand binding, isolated human SAP must be stabilized by a sufficient concentration of serum albumin.

## Functional roles of the pentraxins *in vivo*?

Identification of the roles of human CRP and SAP is complicated by the failure so far to detect any genetic deficiency of either protein: the ultimately informative “experiment of Nature” has not been seen. There are also no common structural variants. Although some extremely rare coding polymorphisms of the *CRP* and *SAP* genes have been noted in genomic studies, the variant proteins that they might encode have not yet been reported. This remarkable conservation suggests that both proteins may have important functions, necessary for survival, presumably in relation to host defense, since this is a major driver of natural selection. However, given the ancient phylogeny of the pentraxins, long antedating acquired immunity, some of these primitive putative “survival” functions are now likely to be redundant.

Our early original studies of pentraxins in other species (25, 26, 41–55) showed that the pentraxin family is phylogenetically ancient with highly conserved sequence homology, secondary, tertiary and quaternary structure as well as calcium dependent ligand binding. Nonetheless, there are major differences between family members in different, even closely related, species. For example, rat SAP (26) has a similar abundance to human SAP (56) (mean (SD, range) concentration, women: 21 mg/l (8. 8-5-5); men: 32 mg/l (7, 12–19, 21–31, 33–52), and neither is an acute phase protein (57). In contrast, mouse SAP baseline concentrations are strain dependent with a ~50-fold range between C57BL/6 (~3–5 mg/l) and DBA (>150 mg/l), and it is a major acute phase reactant rising to >300 mg/l (42). On the other hand mice have low baseline CRP concentrations, ~5–9 mg/l, which rise only twofold in the acute phase response (58). Meanwhile rats have baseline CRP concentrations of ~300–500 mg/l rising 3- to 4-fold in the acute phase response (26). In humans, the median baseline CRP concentration is 0.8 mg/l, with 90% of healthy subjects below 3 mg/l and 99% below 10 mg/l (59). But the concentration can be as low as 50 μg/l (59) and can rise to >500 mg/l at the peak of the acute phase response (60). There are many other variations between species, including behavior as acute phase reactants, precise ligand specificity and the secondary effects of ligand binding: precipitation, agglutination and complement activation. In some species, the hallmark properties of human CRP and SAP are variably distributed between the two pentraxins while neither the dog nor the rabbit even have an SAP gene, although their respective CRP molecules behave rather similarly to human CRP. These findings suggest that the various pentraxins may have different functions in different species and they make it impossible to extrapolate reliably from experimental animal studies to possible functional roles of the pentraxins in humans.

## The challenge of identifying physiological functions of the pentraxins

There have been wide ranging claims, speculations and many evidence-free assertions about pentraxin functions. There are very few robustly definitive observations or experiments. A major weakness in most studies of putative pentraxin functions has been lack of information about the provenance, purity and functional integrity of the CRP and SAP preparations that have been used. Isolation of structurally and functionally intact preparations of these trace plasma proteins, and rigorous demonstration of their quality, are challenging. It is not adequate to use a commercial product or in house preparation without comprehensive characterization. For example, among the many claimed activities is the assertion that the human pentraxins trigger production and secretion of pro-inflammatory cytokines. We have never been able to replicate these reports (36). In order to make definitive observations, we isolated sterile, endotoxin-free, structurally and functionally intact, clinical Good Manufacturing Practice (cGMP) grade human CRP and SAP from pooled normal human plasma of healthy, pathogen free US donors (61). We showed that neither protein had inherent pro-inflammatory effects, either on human peripheral blood mononuclear cells *in vitro* or when administered parenterally to mice or healthy human volunteers *in vivo* (61, 62).

## Functions of human C-reactive protein

Human CRP binds avidly to exposed phosphocholine residues on macromolecules of both autologous and extrinsic origin (22, 63). It then aggregates particulate ligands and precipitates soluble ligands and also triggers classical complement pathway activation (22, 64). Beneficial effects of some of these phenomena may thus underlie the evolutionary persistence of the protein and the highly adaptive regulation of its production in response to injury, infection and inflammation. CRP binds selectively to dead and damaged cells but not to healthy living cells. Phospholipase action on plasma membranes of damaged cells disrupts the normal lipid bilayer, exposing the phosphocholine head groups recognized by CRP. Co-localization of CRP with fixed complement in areas of tissue damage suggests a possible role for CRP in removal of cellular debris from the tissues. However, there is no direct evidence that this function actually operates.

Injection of human CRP into mice at the time of inoculation with virulent pneumococci confers efficient protection against sepsis (65–67). Administration of human CRP after inoculation of the bacteria does not protect. Indeed, all patients with active pneumococcal infections have greatly increased plasma CRP concentrations and abundant circulating human CRP so CRP evidently does not control established pneumococcal sepsis.

In order to study this question further we created pure-line *Crp* gene-deleted C57BL/6 mice using C57BL/6 embryonic stem cells (58). Normally housed CRP deficient mice had normal growth, development, fertility and life span. They did not develop anti-nuclear autoimmunity and responded normally to endotoxin challenge, two processes in which roles for CRP had been proposed (68). However, the CRP-deficient mice were remarkably susceptible to *Streptococcus pneumoniae* infection and were protected by reconstitution with isolated pure human CRP, or by anti-pneumococcal antibodies (58). Autologous mouse CRP is evidently essential for innate resistance to pneumococcal infection before antibodies are produced, probably by clumping the bacteria, limiting their spread and promoting their phagocytosis and destruction by neutrophils. Our findings are consistent with the significant association between clinical pneumococcal infection and non-coding human *CRP* gene polymorphisms which reduce CRP expression (69–71). Deficiency or loss of function variation in CRP may therefore be lethal at the first early-life encounter with this ubiquitous virulent pathogen, explaining the invariant presence and structure of CRP in human adults. Meanwhile, the protective function of mouse CRP against pneumococcal infection is the only function of any CRP to be firmly established so far in the same species.

## Functions of human serum amyloid P component

Continuous treatment for up to several years with the drug, CPHPC (72) (now called miridesap, see below), that persistently depletes circulating SAP by over 90% for as long as the drug is taken, has had no adverse effects (73). Thus, despite its invariant presence, human SAP probably does not have a necessary function in adults.

Our discovery of the avid specific binding of human SAP to DNA (74) and to chromatin (75), where it displaces H1-type histones, thereby solubilizing native long chromatin under physiological conditions, strongly suggested a possible function of human SAP in the *in vivo* handling of exposed DNA and chromatin. Indeed, in *ex vivo* human tissues, both apoptotic cells, which always bear chromatin fragments on their surface, and nuclear debris are always coated with SAP (76, 77). However, our early observation of increased spontaneous anti-nuclear autoimmunity in SAP knockout mice (78) turned out to be limited to the autoimmunity susceptible C57BL/6 strain and not a general effect of SAP deficiency (79). There was no increased autoimmunity, even after autoantigen challenge, with SAP knockout in different mouse strain backgrounds (79). Furthermore, there has been no increased autoantibody production in patients with SAP depletion produced by miridesap (73).

An intriguing possibility is that the avid binding of human SAP to DNA may be the mechanism responsible for the failure of DNA vaccination to be immunogenic in humans. We discovered that there is complete concordance among species tested so far (sub-human primates, dog, rabbit, horse, cow, sheep, pig, goat) between the effectiveness of DNA vaccination and the absence of SAP binding strongly to DNA (unpublished observations). In particular, mice respond well to DNA vaccination and mouse SAP binds DNA very weakly (79). Also transgenic expression of human SAP in mice blocks immune responses to DNA vaccination (80) and this inhibition is completely abrogated by administration of my SAP-depleting drug, CPHPC (miridesap) (72, 81). We therefore lately conducted a preliminary clinical trial, HIV-CORE 003, of SAP depletion by CPHPC (miridesap) in healthy volunteers receiving a DNA vaccine against HIV (82). The results were largely negative although, compared to placebo treated controls, the SAP depleted subjects mounted significantly broader immune responses (82). Further studies of this important question are needed.

SAP is inherently resistant to proteolysis (34) and is also a potent anti-opsonin (83). Its binding therefore “protects” its macromolecular ligands from degradation, whether these are the amyloid fibrils in local or systemic amyloid deposits, or pathogenic bacteria. Indeed those bacterial pathogens to which SAP binds (84), use the bound SAP to shield themselves from the host's phagocytic defenses (83). Thus, for example, SAP knockout mice are more resistant than wild type mice to lethal infection with *Strep. pyogenes* and rough Gram negative bacteria (83). In contrast, SAP deficient mice are more susceptible than wild type controls to lethal infection with smooth Gram negative bacteria, to which SAP does not bind (83). Mouse SAP therefore contributes to innate immunity to some bacterial infections and, although the mechanism is unknown, this is so far the only definite *in vivo* function identified for an autologous SAP.

A host defense role for SAP is potentially consistent with the fact that human SAP binds avidly to Shiga toxin 2 and neutralizes it *in vitro* (85, 86), which led to our demonstration that human SAP protects against cytotoxicity of *E. coli* Shiga toxin 2 for podocytes *in vitro* (87) and against lethality in mice *in vivo* (88). However, we did not find any association between human SAP concentrations and haemolytic uraemic syndrome or antibody titres against toxigenic *E. coli* lipopolysaccharide (88). Although SAP binds many lipopolysaccharides, there is no reproducible evidence that either SAP (83) or CRP (68) protect against their *in vivo* toxicity in mice.

Interestingly, binding of human SAP to the lipopolysaccharide of rough Gram negative bacteria blocks classical complement pathway activation by the endotoxin (89). We had previously discovered (39) that pairs of aggregated SAP molecules, but not single soluble SAP molecules, calcium dependently bind C4-binding protein, a negative regulator protein of the classical cascade. On the other hand, supraphysiological concentrations of human SAP, which undergo calcium dependent autoaggregation, do activate complement. However, the abundant coating of amyloid fibrils with SAP clearly does not activate complement and the *in vitro* observation is therefore probably not relevant *in vivo*.

How the anti-opsonin and “ligand protective” properties of SAP contribute to beneficial functions of the protein remains a matter for speculation. However, in addition to being a circulating plasma protein, human SAP is also a normal constituent of the extracellular matrix; and aggregated human SAP has a highly specific binding interaction with fibronectin (39), another universal matrix glycoprotein. Human SAP is an integral component of the glomerular basement membrane (90) and of the microfibrillar mantle present on elastic fibers throughout the body (91). It is therefore conceivable that the SAP helps to protect the integrity of the structures with which it is associated. Experimental investigation of this concept is challenging. Mouse SAP is not detected in the extracellular matrix of normal mouse tissues and SAP evidently does not have a specific obligatory function since neither dogs nor rabbits have an SAP gene, while horses, which do have an SAP gene, do not express a protein with the same calcium dependent ligand binding specificity as SAP of other species (unpublished observations). Our SAP deficient, gene deleted mice have no phenotype when unchallenged (92), supporting the view that, despite the evolutionary conservation of SAP, its functions may well be redundant in normal health.

## SAP and amyloidosis

**Figure 4:**
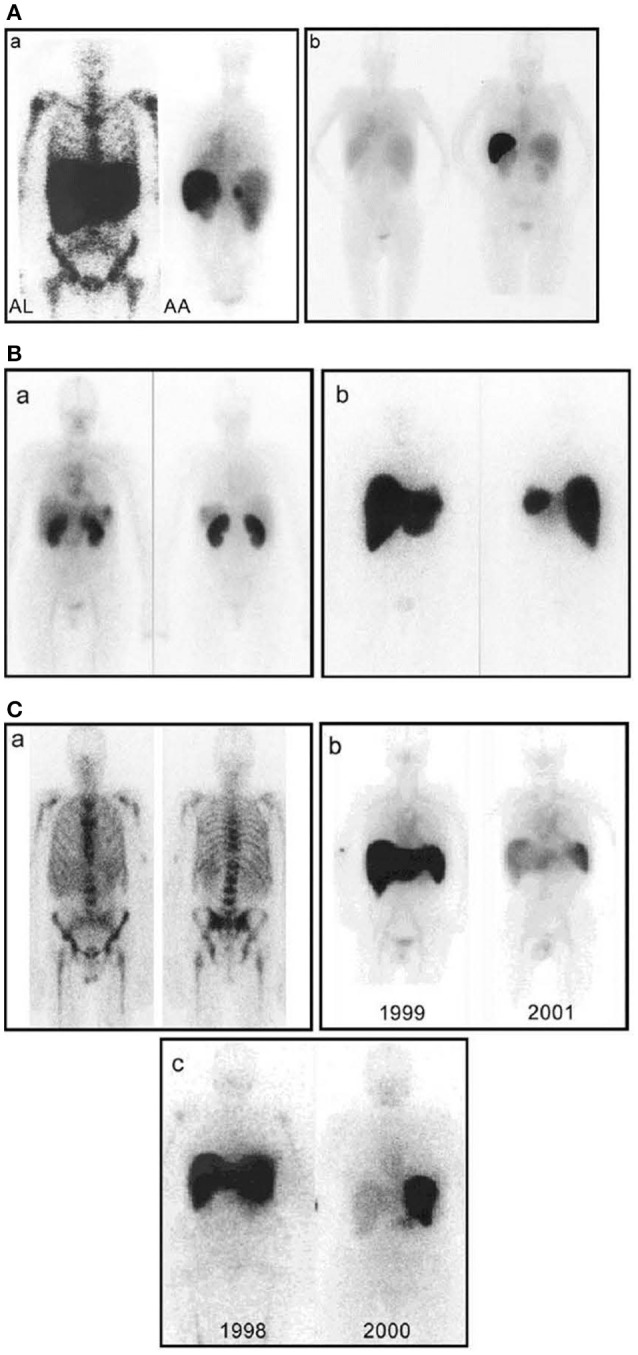
Whole-body scintigraphy with ^123^I-labeled serum amyloid P component in systemic amyloidosis. **(A) (a)** Left: Anterior view of a typical patient with AL amyloidosis showing massive liver and spleen amyloid and the pathognomonic deposits throughout the bone marrow that are not seen in any other type of amyloidosis. Right: Posterior view of a typical patient with AA amyloidosis showing amyloid in the spleen, kidneys, and adrenal. The left adrenal is obscured by the overlying spleen, but the right is clearly visible above the kidney. **(b)** Posterior scans taken a year apart in a patient with longstanding rheumatoid arthritis who suddenly developed AA amyloidosis. The earlier scan (left) is normal; the later one (right) shows heavy splenic and significant renal amyloidosis. **(B) (a)** Anterior (left) and posterior (right) views of a patient with AL amyloid who presented with minor proteinuria and no other clinical or investigational evidence of disease. There is substantial renal amyloid but no scintigraphically detectable de- posits elsewhere. **(b)** Anterior (left) and posterior (right) views of a different patient with AL amyloid who also presented with minor proteinuria and no other clinical or investigational evidence of disease. There is massive amyloid deposition in the liver and spleen. The kidneys are not visualized, probably because the tracer, which distributes according to the amount of amyloid, is all taken up elsewhere. Note that, in contrast to **(a)**, there is no residual tracer in the circulation, indicating a heavy whole-body amyloid load. This patient did not tolerate intensive chemotherapy and developed liver failure. **(C) (a)** Anterior (left) and posterior (right) views of a patient with AL amyloid who presented with multiple fractures over 4 years. X-ray and bone scan were normal but bone biopsy unexpectedly revealed amyloid. No monoclonal gammopathy was identifiable at that time, but bone amyloid is frequent in AL and may be the main clinical feature. **(b)** Serial anterior views showing regression of AA amyloidosis in a juvenile rheumatoid arthritis patient treated with chlorambucil, in whom the SAA concentration was suppressed to <10 mg/l. **(c)** Serial anterior views showing regression of AL amyloidosis in a patient treated with high-dose melphalan and stem cell rescue. [From Pepys (100) with permission of Annual Reviews].

My discovery of calcium dependent ligand binding by SAP to agarose (9, 21) enabled our demonstration that the analogous binding of SAP to amyloid fibrils is responsible for the universal presence of SAP in all amyloid deposits of all types in humans (93). We formally demonstrated that the circulating SAP is the precursor of amyloid P component (AP) in amyloid deposits (94). This led directly to my use of radiolabelled SAP as an amyloid specific tracer *in vivo* (95, 96) and the invention of SAP scintigraphy and metabolic studies (97–99). The ability to image amyloid throughout the whole body in patients with systemic amyloidosis and thus, safely and non-invasively, localize and quantify amyloid deposits, has made major contributions to understanding the natural history of amyloidosis and its response to therapy (100) (Figure 4). Once the scan became available, the Immunological Medicine Unit at the Royal Postgraduate Medical School soon became the *de facto* national referral center for amyloidosis patients in the UK. In 1999, when I moved with my team to the Royal Free Campus of University College Hospital, the UK Department of Health funded us as the NHS National Amyloidosis Centre to provide diagnostic and management advice for the whole national caseload (www.ucl.ac.uk/amyloidosis/ and www.amyloidosis.org.uk). The Centre now sees over 4,000 amyloidosis patients per year, follows the world's largest and most diverse cohort of such patients and has conducted about 40,000 SAP scintigraphy studies since 1988 with no adverse effects.

The observation of calcium dependent ligand binding by SAP (9, 21) also led toward potential new treatments. My serendipitous finding in 1983 that widely differing amounts of SAP bound to different batches of Sepharose led to the discovery that SAP binding correlated precisely with the pyruvate content of the agarose. Pyruvate is a variable trace component present as the cyclic acetal of β-D-galactopyranose in agarobiose (101). We synthesized the monosaccharide, methyl 4,6-*O*- (1-carboxyethylidene)-β-D-galactopyranoside (M*O*βDG) and showed that it completely blocked and reversed the binding of SAP to all its known ligands, crucially including pure protein ligands and amyloid fibrils with no carbohydrate present (101). These seminal results enabled localisation of the calcium dependent ligand binding site in SAP when we solved its 3D X-ray crystal structure (28), and also led to a new therapeutic approach. Although we did not then know the role, if any, of SAP in pathogenesis of amyloidosis, the finding that MOβDG could remove all the SAP bound in amyloid deposits suggested an approach to disrupting the deposits and promoting their clearance (102).

We subsequently showed that AP in amyloid is identical to its SAP precursor in the plasma and remains completely intact despite very prolonged residence in the tissue deposits (103). The plasma half-life of SAP in normal healthy subjects is ~24 h whilst the half-life of SAP in visceral amyloid deposits is ~30 days (99). Furthermore, binding of SAP to amyloid fibrils *in vitro* mutually protects the fibrils and the SAP from degradation by proteases and phagocytic cells (104). SAP, although itself rather resistant to proteolysis, is not a protease inhibitor. It protects the fibrils only when it is actually bound to them (104).

Amyloid fibrils are readily digested by proteases and ingested and degraded by phagocytic cells *in vitro*. In contrast, *in vivo*, systemic amyloid deposits are almost entirely ignored by the normally highly efficient cellular and molecular mechanisms for clearance of extracellular debris from the tissues. The reasons for this are unknown but, in view of our discovery that bound SAP protects amyloid fibrils from degradation *in vitro*, I proposed that it might do the same thing *in vivo*. I hypothesized that the universal, ubiquitous coating of SAP on amyloid fibrils *in vivo* protects them from clearance and removal (104). I claimed that stripping of bound SAP, and prevention of SAP binding, would enable amyloid deposits to be recognized as abnormal and therefore phagocytosed and degraded, leading to amyloid removal (105). We then went on to create the first SAP knockout mice and to show that, although it was possible to induce systemic AA amyloidosis in them, it took longer than in wild type mice and the deposits were smaller (92). SAP was thus validated as a therapeutic target. Meanwhile SAP was also shown to promote amyloid fibril formation from soluble precursors *in vitro*^3^, apparently by binding to and stabilizing protofibrillar aggregates (109–111).

**Figure 5:**
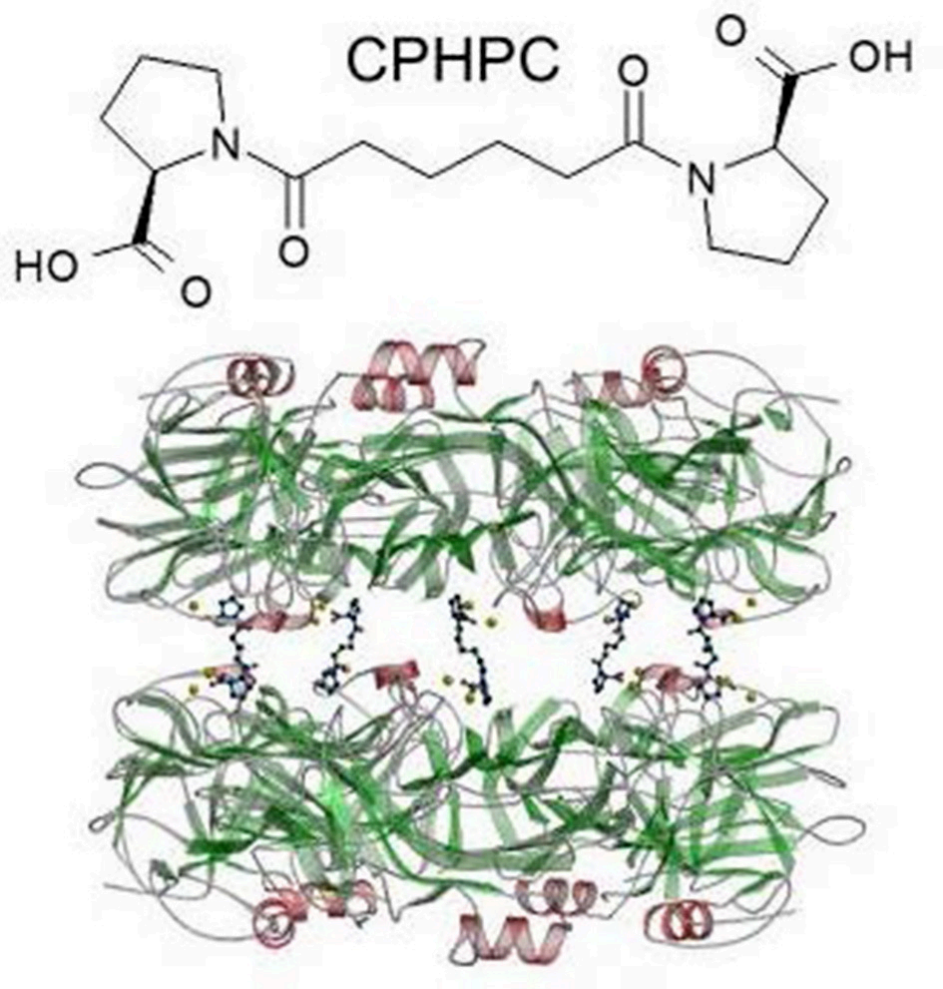
Structure of CPHPC (miridesap) and its complex with human SAP. The palindromic bivalent structure of (R)-1-[6-[(R)-2-carboxy-pyrrolidin-1-yl] -6-oxo-hexanoyl]pyrrolidine-2-carboxylic acid (CPHPC), now known by its WHO INN, miridesap, is shown above. Below is the 3D X-ray crystal structure of the SAP-drug complex which is also the structure of the complex in solution (112). [From Pepys et al. (72) with permission of Macmillan Publishers Ltd].

I invented a high throughput screen for inhibitors of SAP binding to amyloid fibrils (105) and in the late 1990s, I persuaded Roche to use it to explore their compound library. With the help of some fortuitous serendipity, this swiftly led to the creation of a drug candidate, (R)-1-[6-[(R)-2-carboxy-pyrrolidin-1-yl]-6-oxo-hexanoyl]pyrrolidine-2-carboxylic acid, which I abbreviated as CPHPC, a palindromic acronym for a palindromic molecule^4^ (72). Binding of SAP to amyloid fibrils and all its other known ligands is inhibited by CPHPC because SAP binds to the drug in a complex composed of two pentameric SAP molecules cross linked face to face by five of these bivalent hexanoyl bis(D-proline) molecules (72) (Figure 5). Each D-proline head group is located in the calcium dependent ligand binding pocket of a protomer. Although N-acetyl D-proline is only weakly bound by SAP, with Kd ~15 μM, the cross linking of pairs of SAP molecules by five CPHPC molecules forms a very stable complex with Kd ~10 nM due to the avidity gain of multivalency. In the SAP-CPHPC complex, all the calcium dependent ligand binding sites are occupied and the ligand binding “B” face of the disc-like SAP molecules is also occluded (72, 112) (Figure 5).

**Figure 6:**
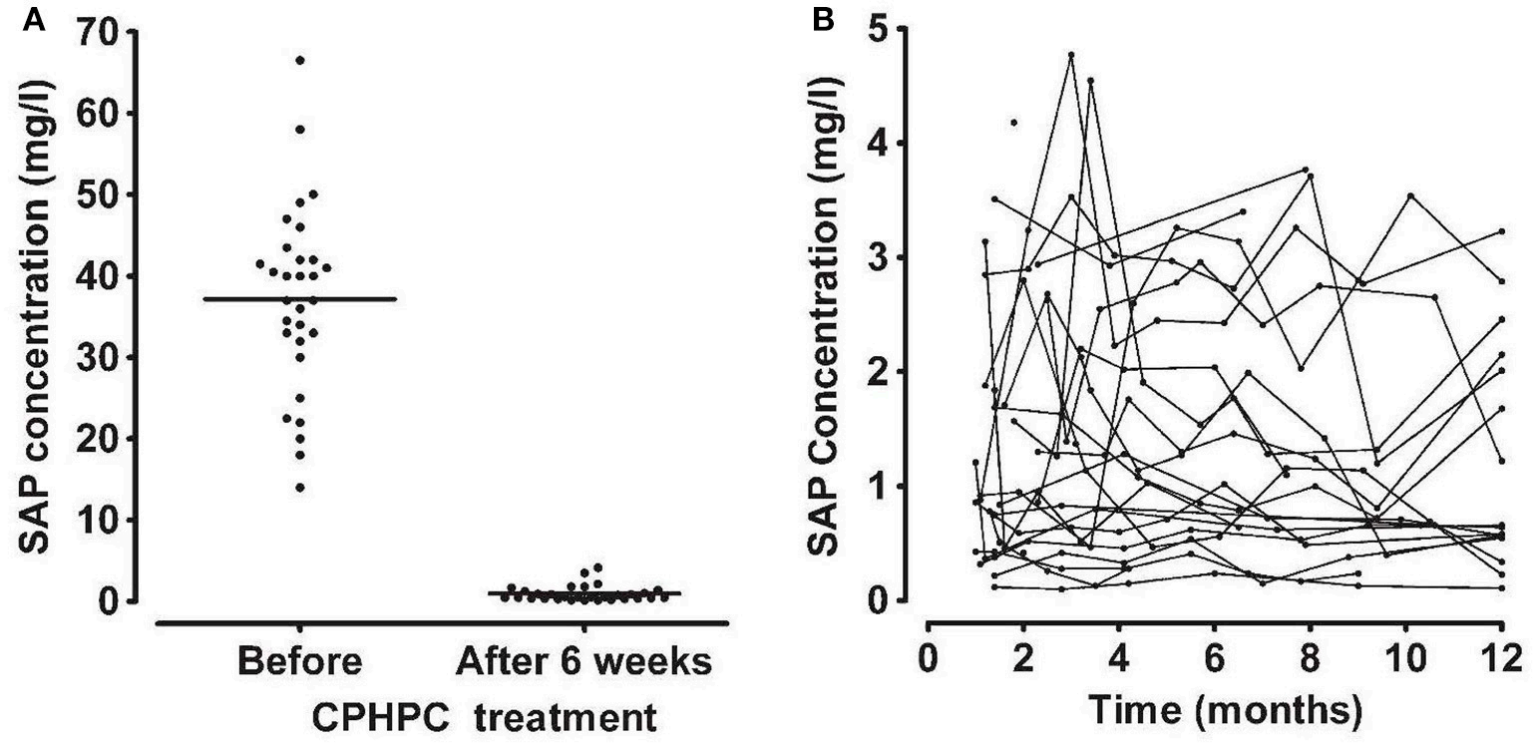
Depletion of circulating SAP by CPHPC (miridesap) in patients with systemic amyloidosis. **(A)** Serum concentration of SAP immediately before and 6 weeks after starting daily treatment with CPHPC. **(B)** Sustained depletion of SAP throughout CPHPC treatment. Each line shows the results of serial measurements in an individual patient. Note different scale for SAP concentration compared to **(A)**. From Gillmore et al. (73) with permission of Blackwell Publishing Ltd). In patients without systemic amyloidosis and the associated massive extracellular load of SAP (98), CPHPC (miridesap) treatment reduces plasma SAP concentration to much lower values, for example, mean (SD) 0.25 (0.16) mg/l, in our 5 patients with Alzheimer's disease (112).

Work toward clinical testing in humans proceeded rapidly but, shortly before the first in human study, Roche stopped their development and handed the project over to us. Our first administration of CPHPC to humans immediately revealed that the drug produced very rapid and almost complete depletion of SAP from the circulation that persisted for as long as the drug was given (72, 73) (Figure 6). We showed that this resulted from the instant clearance of the SAP-CPHPC complex by the liver (72), where the SAP was promptly destroyed whilst the CPHPC, which is not metabolized at all, is released and swiftly excreted, mainly in the urine and to a smaller extent in the bile. The invention of CPHPC and the novel, and so far unique, pharmacological mechanism, by which a small molecule drug produces a targeted knockout of a pathogenic plasma protein, was recognized by the American Chemical Society as one of the medicinal chemistry highlights of 2002. CPHPC itself and prolonged SAP depletion were both well tolerated with no adverse effects other than mild transient stinging at sites of subcutaneous injection of the drug (73). However, the treatment did not promote regression of amyloid deposits from the tissues of patients with systemic amyloidosis. Depletion of circulating SAP removed much but never all SAP from its binding to amyloid, despite months of CPHPC treatment (73). This reflects a combination of factors that cannot be overcome: the avidity of binding of SAP to amyloid fibrils, the continuous production of new SAP by the liver, and the rapid excretion of CPHPC. In addition, crucially, the avid binding of SAP to CPHPC requires simultaneous binding of multiple D-proline head groups by pairs of SAP molecules. Complete elution of SAP from amyloid deposits therefore requires the presence of ~1 mM CPHPC, an extremely high concentration that is not attainable *in vivo* despite the excellent tolerability of the drug. Something more was required to clear amyloid.

**Figure 7:**
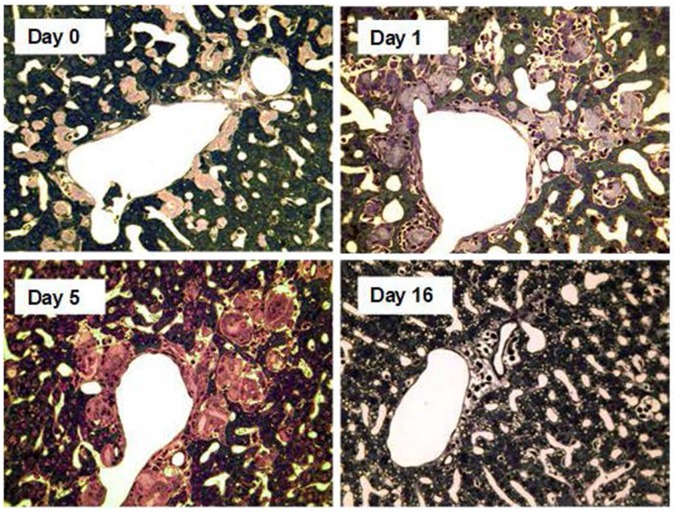
Amyloid clearance mediated by macrophage derived multinucleated giant cells after depletion of circulating SAP followed by treatment with anti-SAP antibody. Thin sections of liver stained with toluidine blue from AA amyloidotic human SAP transgenic mice treated with CPHPC (miridesap) to deplete circulating SAP followed by anti-SAP antibody to target residual SAP in the amyloid deposits. Control mouse, not treated with anti-SAP antibody, show abundant amorphous pink-stained amyloid deposits, with the characteristic absence of any surrounding inflammatory reaction or cellular infiltrate. One day after anti-SAP antibody treatment there is intense, predominantly mononuclear cell infiltration in and around the amyloid. Five days after anti-SAP-antibody treatment there is fusion of macrophages to form multinucleated giant cells surrounding and infiltrating the deposits and containing large masses of ingested amyloid undergoing degradation. At 16 days there is complete elimination of amyloid deposits with no residual cellular infiltrate and restoration of normal tissue architecture. [From Bodin et al. (114) with permission of Macmillan Publishers Ltd].

Phagocytosis and degradation by macrophages is the most important mechanism for removal of autologous debris and extrinsic materials from the extracellular space of the tissues. It is potently engaged by antibody mediated complement activation. In 2005, I realized that the residual SAP left in amyloid deposits, after depletion of the circulating SAP by CPHPC, could be used as a target for anti-SAP antibodies that would trigger amyloid removal (113). We tested the idea in human SAP transgenic mice in which we had induced systemic AA amyloidosis (114). Circulating human SAP was depleted with CPHPC and the mice then received a single dose of either sheep polyclonal anti-human SAP antibody or of normal control sheep IgG. There were no discernible adverse effects. Within 2 weeks almost no amyloid was detectable in the anti-SAP treated animals, compared to the unchanged massive amyloid load in the controls (114). Both classical complement pathway activation and macrophages were necessary and amyloid clearance was effected by multinucleated, macrophage derived, giant cells which surrounded, engulfed and destroyed the amyloid within days of antibody administration (114) (Figure 7). Depletion of SAP from the plasma and extracellular fluid is obviously essential, before administration of the anti-SAP antibody, so the proposed treatment is an obligate therapeutic partnership, not just a combination of two different drugs. Suitable avid, complement activating mouse monoclonal anti-human SAP antibodies were as effective as the xenogeneic polyclonal antibody (113), enabling potential clinical implementation with humanized antibody. In 2009, the invention was licensed to GlaxoSmithKline (GSK) for clinical drug development.

**Figure 8:**
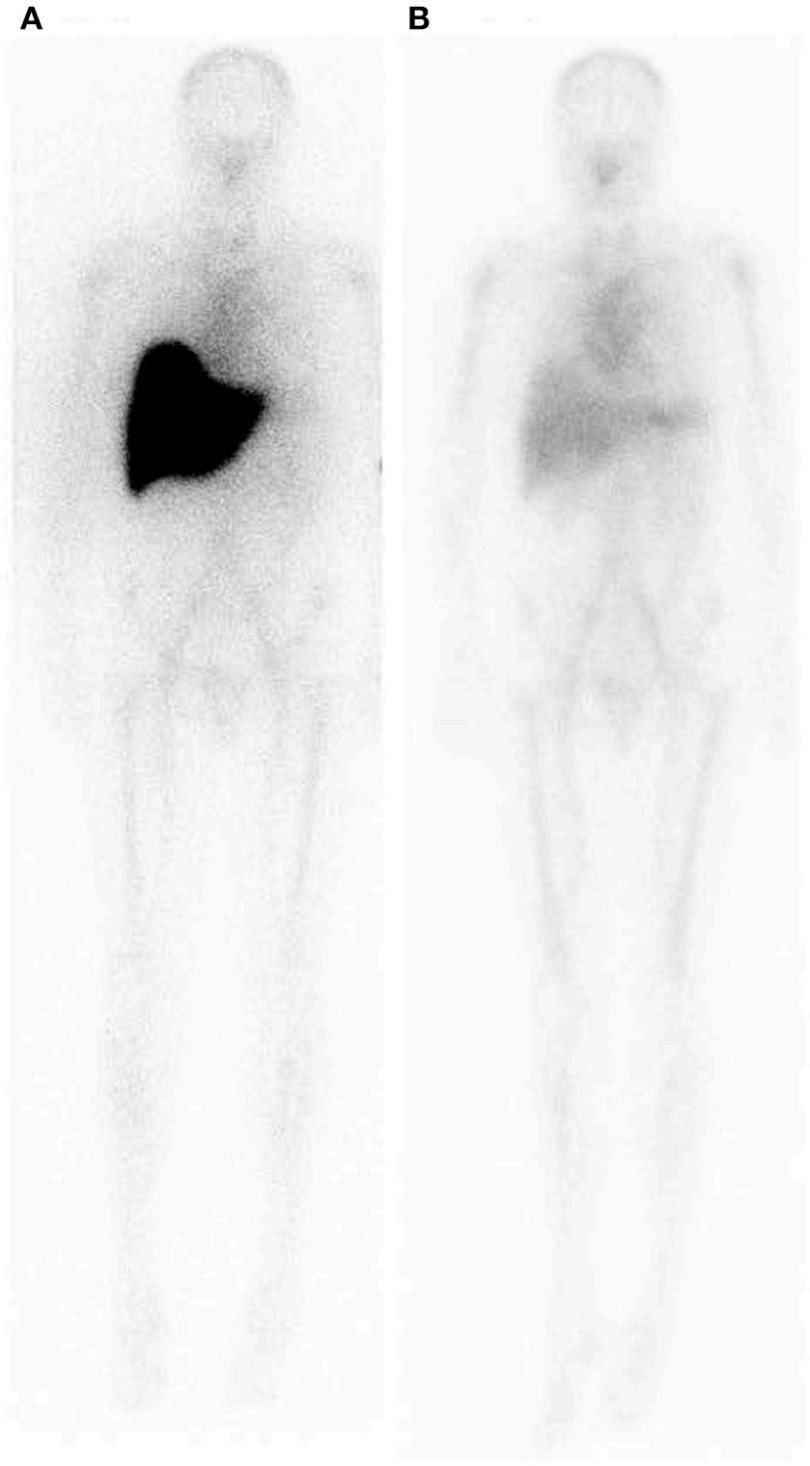
Whole body scintigraphy with ^123^I-labeled serum amyloid P component in a patient with systemic amyloidosis before and after depletion of circulating SAP followed by treatment with anti-SAP antibody. **(A)** Scan immediately before treatment. **(B)** Scan 42 days after single dose of dezamizumab (fully humanized monoclonal anti-human SAP antibody) infused following depletion of circulating SAP with miridesap. The heavy load of amyloid in the liver has been dramatically reduced. [From Richards et al. (115) with permission of Massachusetts Medical Society].

GSK fully humanized our optimal mouse monoclonal antibody and the first in human phase 1 study in patients with different types of systemic amyloidosis, starting in 2013, demonstrated unprecedented removal of visceral amyloid, with progressive removal after serial antibody doses (115, 116) (Figure 8). The antibody caused moderate infusion reactions and higher antibody doses produced skin rashes but there was no disturbance of organ function, even in heavily amyloidotic organs. Indeed abnormal liver function tests returned toward normal in all patients as their amyloid load was reduced (115, 116). All amyloid reducing doses of anti-SAP antibody produced transient early acute phase responses and dramatic depletion of plasma complement C3 concentration, consistent with activation of the same mechanism as we characterized in mice (115, 116). In 2017, the two drugs received their WHO International Non-proprietary Names (INN), miridesap for CPHPC and dezamizumab for the humanized monoclonal anti-SAP antibody, and the encouraging phase 1 results led to the current GSK phase 2 trial in patients with cardiac amyloidosis.

## SAP, Alzheimer's disease and cerebral amyloid angiopathy

Miridesap was intended from the outset to target SAP associated with the Aβ amyloid deposits in the brain and cerebral vasculature in Alzheimer's disease, as well as for systemic amyloidosis. Human SAP is synthesized only by the liver. As we had predicted, our initial, preliminary, clinical study in Alzheimer's disease confirmed that depletion of circulating SAP also completely removed SAP from the cerebrospinal fluid (112). Our subsequent study in a triple transgenic, human SAP expressing, mouse model of human Alzheimer's disease, confirmed that miridesap does indeed achieve the desired “molecular dissection” of Alzheimer's disease neuropathology by removing all SAP from cerebral amyloid deposits (117). This contrasts with the failure of miridesap to removal all SAP from the enormously more abundant visceral amyloid deposits in systemic amyloidosis (73), and, encouragingly, should enable the original SAP removal hypothesis to be tested with respect to cerebral amyloid. This is one of the goals for our current “Depletion of serum amyloid P component in Alzheimer's disease” (DESPIAD) phase 2b clinical trial of miridesap. We also hope to study SAP depletion in cerebral amyloid angiopathy (118), the most prevalent form of clinical amyloidosis.

However, there is another rationale for SAP depletion in these brain diseases. Human SAP is directly cytotoxic for cerebral neurones, *in vitro* and *in vivo*, causing death by apoptosis (119–123). The SAP enters the cells, tracks to the nucleus, presumably via the nuclear localisation sequence present in pentraxins (124), enters the nucleus and then binds to chromatin, as we first demonstrated (75, 125). We have lately confirmed and extended (unpublished observations) an original preliminary report (126) that individuals with dementia have a higher brain content of SAP than individuals without dementia, regardless of the presence of Alzheimer's disease neuropathology. The results are consistent with a possible direct pathogenetic role of SAP in dementia, unrelated to the role of SAP in amyloid. Detection of potential benefit from abrogation of direct SAP neurotoxicity is the other major goal of the DESPIAD trial.

## Routine clinical measurement of CRP

My initial measurements of serum CRP concentration in 1975 swiftly showed that CRP was an excellent marker of Crohn's disease, closely reflecting extent and activity much better that any other single measurement (127). Our subsequent work confirmed and extended the results (128–130). I also made the striking discovery that the CRP response in ulcerative colitis, which, in 1975, also had not previously been reported, was completely different from Crohn's disease. Despite even severe, extensive, active ulcerative colitis, the circulating CRP concentration was generally modestly increased if at all (127, 128, 130). The unexpected, surprising, original observation of a marked difference in the CRP response to two rather similar disease processes, both with extensive inflammatory activity and tissue damage, initiated my lifelong interest in the clinical significance and utility of CRP assays.

Initially we studied the behavior of CRP as an acute phase reactant in a very broad range of different conditions, in well characterized series of patient, thereby establishing the optimal use of CRP in routine clinical practice (Tables 1, 2). Commercial instrument based, rapid quantitative CRP immunoassays emerged in the early 1980s and modern high throughput automatic clinical chemistry analysers followed. In 1983, the World Health Organization invited me to create the First International Reference Standard for Immunoassay of C-reactive protein 84/506 (131). It remains the primary standard for all commercial clinical measurement of CRP. I also provided all the CRP for the major international secondary standards, the IFCC CRM470 and the ERM DA470 and ERM DA472. By virtue of my uniquely broad clinical experience with CRP measurement, and the expertise I had acquired in very large scale isolation and purification of human CRP to provide standards and calibrators, I played a substantial role in development of routine clinical CRP testing worldwide, working closely with major diagnostics companies. As recently noted by the EU SCIENCE HUB, the European Commission's science and knowledge service, “C-reactive protein (CRP) is one of the most important analytes in clinical chemistry.” I have comprehensively reviewed elsewhere the scientific and clinical basis for routine use of CRP measurements (60, 132) (Tables 1, 2).

**Table 1:** Human CRP responses in different diseases.

**Major CRP acute-phase response**	
Infections	Bacterial
	Systemic/Severe fungal, mycobacterial, viral
Allergic complications of infection	Rheumatic fever
	Erythema nodosum
Inflammatory disease	Rheumatoid arthritis
	Juvenile chronic arthritis
	Ankylosing spondylitis
	Psoriatic arthritis
	Systemic vasculitides
	Polymyalgia rheumatica
	Crohn's disease
	Familial Mediterranean fever
	Cryopyrin-associated periodic syndromes
Necrosis	Myocardial infarction
	Stroke
	Tumor embolisation
	Acute pancreatitis
Trauma	Surgery
	Burns
	Fractures
Malignancy	Lymphoma
	Carcinoma
	Sarcoma
**Modest or absent CRP acute-phase response**	
	Systemic lupus erythematosus
	Scleroderma
	Dermatomyositis
	Ulcerative colitis
	leukemia
	Graft-vs.-host disease

**Table 2:** Routine clinical uses of CRP measurement.

**Screening test for organic disease**
**Assessment of disease activity in inflammatory conditions**
Juvenile chronic (rheumatoid) arthritis
Rheumatoid arthritis
Ankylosing spondylitis
Psoriatic arthropathy
Systemic vasculitides
Polymyalgia rheumatica
Crohn's disease
Rheumatic fever
Familial Mediterranean fever
Cryopyrin-associated periodic syndromes
Acute pancreatitis
**Diagnosis and management of infection**
Most systemic/severe bacterial, mycobacterial, viral and fungal infections
Response to antimicrobial treatment
Bacterial endocarditis
Neonatal septicaemia and meningitis
Intercurrent infection in systemic lupus erythematosus
Intercurrent infection in leukemia and its treatment
Postoperative complications including infection and thromboembolism
**Differential diagnosis/classification of inflammatory disease**
Systemic lupus erythematosus vs. rheumatoid arthritis
Crohn's disease vs. ulcerative colitis

## CRP as a therapeutic target

Our original 1994 report identified for the first time the association between acute phase responses and adverse prognosis in acute coronary syndromes (133). Our 1997 epidemiological work on CRP in patients with angina (134), and studies by others in general populations, identified an association between increased baseline values of CRP and future incidence of cardiovascular disease. The association initially seemed potentially consistent with a pathogenic role for CRP in atherosclerosis and stimulated very widespread clinical interest in CRP, particularly as it was so easy to measure. An avalanche of epidemiological and experimental observations followed, purporting to show that CRP is a pro-atherogenic risk factor for cardiovascular disease. We were initially enthusiastic but it was soon clear that the early observational epidemiology cohorts had grossly overestimated the significance of the association. They included large total numbers of subjects but only small numbers of cardiovascular disease events, and their interpretation was then flawed by remorseless conflation of the overestimated association with causality. Poorly controlled experimental work purporting to show atherogenic activities of CRP was also badly flawed by use of uncharacterized and often contaminated CRP preparations. It soon became clear there was no evidence for causality of CRP in cardiovascular disease, as detailed in our extensive critical reviews (35, 135, 136). Appropriately large scale observational epidemiology firmly established that baseline CRP values are actually only a very modest risk marker for cardiovascular disease (137, 138) and Mendelian randomization studies proved that CRP itself is definitely not a causative risk factor (139). In addition to many unequivocally negative experimental *in vitro* and *in vivo* studies (35, 135, 136), we finally showed that direct infusion of pharmaceutical grade authentic cGMP human CRP had no pro-inflammatory effects in healthy volunteers (62) in contrast to the pro-inflammatory effect of recombinant CRP made in *E. coli*!

In contrast to the now discredited idea that CRP is pro-atherogenic, the evidence for a role of CRP in exacerbation of pre-existing ischemic and other tissue injury is robust. Complement has long been known to be responsible for the inflammatory neutrophil infiltrate that characterizes experimental acute myocardial infarction (140) and it had been speculated that CRP, via its capacity to activate complement after binding to its ligands *in vivo*, might exacerbate tissue damage (141–145). In 1999 we were the first to actually demonstrate this *in vivo*, using the rat acute myocardial infarction model (24). Although rat CRP circulates at very high concentration in normal healthy animals, rat CRP does not activate rat complement whereas human CRP activates both human and rat complement (26). Rat thus provide an excellent model for investigation of the effects of human CRP in humans. Administration of isolated pure human CRP to rats following ligation of the coronary artery substantially increased the size of the resulting myocardial infarct and human CRP was co-deposited with rat complement on and around the infarcted tissue (24). Crucially, the exacerbation of injury by human CRP was completely abrogated by prior depletion of C3 using cobra venom factor (24). The CRP effect was thus totally complement dependent. We subsequently showed that human CRP also increased cerebral infarct size in the rat middle cerebral artery occlusion model (146).

Having identified and validated human CRP as a therapeutic target, we designed novel bis(phosphocholine)-alkanes as inhibitors of ligand binding by human CRP *in vivo*. These ligands for CRP were based on our knowledge of the 3D X-ray crystal structure of the CRP-phosphocholine complex (31) and our experience with miridesap, hexanoyl-bis(D-proline), the SAP inhibitor drug (72). We showed that bis(phosphocholine)-hexane (32) and bis(phosphocholine)-octane (unpublished) completely abrogated the enhancement of tissue damage caused by human CRP in the rat acute myocardial infarction model. Binding of human CRP to these compounds inhibits CRP binding to other ligands, though it does not accelerate clearance of CRP from the circulation as miridesap does with human SAP.

Exacerbation by CRP of ischemic and inflammatory tissue injury in various different animal models has been independently confirmed by other groups. Abrogation of the pathogenic CRP effect has also been replicated with our compound, bis(phosphocholine)-hexane, and by suppression of CRP production with antisense oligonucleotides, and by using CRP apheresis to remove circulating CRP (147–154).

The cross linking of pairs of CRP molecules by five bis(phosphocholine)-alkane molecules markedly stabilizes the non-covalent homopentameric assembly of native human CRP, preventing denaturation and the release of protomers. However, in the absence of calcium or of calcium dependent ligand binding, denatured CRP can dissociate *in vitro* to release free protomers, so-called monomeric or “mCRP,” that bear specific neoepitopes. Based on *ex vivo* immunohistochemical detection of these epitopes, it has been asserted that mCRP products of CRP denaturation mediate the pro-inflammatory effects of CRP *in vivo* (155). Inhibition by bis(phosphocholine)-hexane of CRP-mediated inflammation has then been attributed exclusively to stabilization of native CRP (155), curiously ignoring our unequivocal demonstration of the absolute complement dependence of the pro-inflammatory actions of human CRP *in vivo* (24). Fortunately this oversight and mechanistic disagreement have no practical importance as the avid binding of our palindromic CRP inhibitor ligands, designed to prevent CRP-mediated complement activation *in vivo*, inevitably also robustly stabilizes the native pentameric CRP structure.

The bis(phosphocholine)-alkanes are well tolerated and would have been suitable for development as infusional drugs but it was not possible to synthesize and purify them at scale. We have therefore designed new, different and more potent inhibitors of CRP binding and are currently working toward candidate selection for clinical development. Clinical observations are consistent with the experimental evidence that high circulating CRP concentrations exacerbate pre-existing tissue damage. For example, higher CRP values during and after acute myocardial infarction are strongly associated with poor prognosis overall, including more extensive myocardial injury, impaired cardiac function and progression to heart failure (156). The same is true in a wide range of other tissue damaging ischaemic, inflammatory, infective, traumatic and malignant conditions. There are thus likely to be many indications for therapeutic use of CRP inhibitor drugs.

## Conclusions

The range of physiological and pathophysiological roles of the pentraxins remains incompletely understood. Their gene and amino acid sequences, and very characteristic molecular assembly, are highly conserved in phylogeny and there are no human genetic deficiencies or even isoforms, and yet there are major differences in behavior and properties between even closely related species. The pentraxins thus display a fascinating, and so far unexplained, mixture of conservation and plasticity in a single protein family. However, regardless of their normal roles, both human CRP and SAP have become extremely useful in clinical diagnosis and monitoring of disease. CRP assay is one of the most widely used clinical chemistry tests and SAP scintigraphy has transformed understanding and optimal management of systemic amyloidosis. Importantly, human CRP and SAP are also therapeutic targets for which the design and development of potential new medicines are making exciting progress.

## Ethics statement

No new work on human or animal subjects is included in the article. All work on human and animal subjects that is mentioned in the article was conducted with full ethical approval current at the time of publication, as detailed in the original source literature which is cited in the references.

## Author contributions

MP conceived and wrote the paper and takes full responsibility for all observations, results and opinions expressed therein.

### Conflict of interest statement

MP is the inventor on patents for miridesap (CPHPC) and miridesap plus anti-SAP antibody: WO 03/013508 A1, “Therapeutic agent for depletion of an unwanted protein population from the plasma”; WO/2009/155962, “Use”; and WO/2009/000926, US7910106 B2, and US9192668 B2, “Combinations of SAP depleting agents and anti-SAP antibodies.” MP founded and owns shares in Pentraxin Therapeutics Ltd, the University College London spinout company that owns these patents and EP 0915088B1 A1, “D-proline derivatives.”

## Footnotes

^1^Serendipity: “making discoveries, by accident and sagacity, of things not sought” *Horace Walpole, 28 Jan 1745*.

^2^Pentraxin is a splendid name but I confess to having misleadingly tried to change it. In 1983, based on advice by Greek colleague who wrongly thought that the etymology was from “*penta”* and “*axin,”* I suggested, incorrectly, that the name should be “pentaxin” (20). Alex Osmand corrected me - *mea culpa*.

^3^In the artefactual, non-physiological, absence of calcium, SAP inhibits the formation of Aβ amyloid fibrils *in vitro* (106). Indeed, we subsequently found that human SAP has classical chaperone properties in protein refolding assays (37, 107). The activity is calcium independent, does not involve ligand binding by the SAP and is apparently mediated by the “A” face of the molecule (37) but it is not clear whether and how it might operate *in vivo*.

^4^The high throughput screen of 100,000 compounds identified a small number of hits, the most attractive of which was one of the four disastereoisomers of captopril. Captopril itself and a third isomer were inactive but the fourth was more active than the original hit. When the actual material being tested was analyzed, it was found that the active substance was no longer the compound itself, with a free sulphydryl group, but a disulphide bonded covalent dimer. We had then lately reported the 3D X-ray crystal structure of the complex of SAP with dAMP, in which pairs of SAP molecules were cross linked face to face by hydrogen bonding between each dAMP molecules held in the calcium dependent ligand binding pockets of each SAP protomer (108). It was therefore obvious that similar cross linking of SAP molecules by a palindromic covalent structure would be a more potent inhibitor of SAP binding to other ligands than just single univalent ligands potentially occupying individual protomer binding sites. The disulphide dimer of the original hit was then synthesized and, as we had predicted, it was the most potent inhibitor. Further medicinal chemistry to replace the sulfur atoms with the hexanoyl chain yielded (R)-1-[6-[(R)-2-carboxy-pyrrolidin-1-yl]-6-oxo-hexanoyl]pyrrolidine-2-carboxylic acid (CPHPC).
